# Editorial: Pediatric wounds and tissue engineering/regeneration

**DOI:** 10.3389/fped.2026.1815970

**Published:** 2026-09-10

**Authors:** Kim Schilders

**Affiliations:** Alliance of Dutch Burn Care, Beverwijk, Netherlands

**Keywords:** pediatric surgery, pediatric wounds, tissue engineering, tissue regeneration, wound healing

Pediatric wounds remain a major clinical and societal challenge. Factors such as growth, physiology and anatomy make wound healing in children unique compared to wound healing in adults (1, 2). This is for instance evident in burn wounds, which tend to be more severe in children due to their thinner skin. This has a major effect on the quality of life of these patients, since these wounds often result in scar formation (3). Also, the pediatric immune system may respond differently compared with that of adults, which influences wound healing (2). All these factors should be taken into consideration concerning surgical interventions and treatment strategies (4).

The selection of surgical interventions depends on several factors such as depth, size and location of the wound. Furthermore, the distinct anatomical and physiological characteristics of pediatric patients must be carefully considered when planning surgical interventions. Important factors to take into consideration are scar management and growth potential, but also psychological support (5, 6).

In this Research Topic we focused on the treatment of wounds in children. The four articles included in this collection—by Shang et al., Niu et al., He et al., and Lin et al.—highlight the diversity of contemporary challenges in pediatric wound management and show how multidisciplinary progress can directly improve outcomes for children.

While regenerative medicine holds promise, Niu et al. remind us of the dangers posed by unregulated wound treatments. Their striking case report describes a 1-year-8-month-old boy who suffered acute liver failure after his burn wound was treated with a local folk remedy—a practice still widely used in parts of China due to beliefs about its anti-scar properties. Toxicological analysis revealed extremely high concentrations of nickel, chromium, tin, lead, and mercury in both the remedy and the child's wound and blood samples, confirming transdermal absorption through burned skin.

This case underscores the significant permeability of pediatric skin and the increased systemic risk in infants and toddlers. Beyond the immediate hepatotoxicity, such exposure may have long-term neurodevelopmental consequences. Niu et al. reinforce the critical importance of public education, early suspicion of toxic exposure, and strong guidance toward evidence-based burn care. Their report also supports the broader theme of this Research Topic: safe wound healing requires both scientific innovation and community awareness.

Among the studies in this Research Topic, the work by Shang et al. is particularly illustrative of how biologic agents can modulate pediatric wound repair. Their retrospective analysis evaluated autologous platelet-rich plasma (PRP) as an adjunct to tubularized incised plate (TIP) urethroplasty in 103 children with penile hypospadias. PRP, rich in growth factors such as PDGF, VEGF, and TGF-*β*, has the potential to accelerate epithelialization, promote angiogenesis, and improve tissue organization. Shang et al. demonstrate that intraoperative PRP injections significantly reduced early postoperative pain, improved day-7 wound healing, and led to markedly fewer complications, including fistulas and strictures, compared to standard urethroplasty alone. Their findings emphasize not only the reparative potential of growth factor-based therapies but also the need for rigorous evaluation of such biologics in pediatric populations.

The case report by He et al. addresses a rare but educational scenario: accessory spleen hyperfunction appearing seven years after splenectomy in a child with hereditary spherocytosis. Although not a wound-healing study *per se*, this report demonstrates how surgical decisions in childhood—such as preserving accessory spleens to maintain immune function—can have long-term consequences requiring re-operation. The authors detail a technically challenging laparoscopic excision of two large accessory spleens and multiple implanted splenic nodules. The surgical complexity, including dense adhesions and significant vascularization, reflects the regenerative and adaptive capacity of pediatric tissues. Their literature review also highlights the ongoing debate on partial splenectomy vs. total removal in young children, and how postoperative physiology can influence future wound care, immune capacity, and recovery.

In their randomized controlled trial, Lin et al. provide valuable evidence comparing ultrasound-guided caudal block (CB) with dorsal penile nerve block (DPNB) in children undergoing concealed penis correction surgery. They demonstrate that caudal block leads to significantly lower analgesic requirements within 24 h, reduced early postoperative pain, fewer intraoperative adverse events such as tachycardia or body movement, and higher satisfaction from both parents and surgeons. The study highlights how ultrasound guidance and regional anesthesia techniques can enhance safety and effectiveness in pediatric surgery. Although postoperative wound healing was not the primary outcome, improved pain control is well known to support favorable healing trajectories and reduce complications.

In conclusion, wound healing in children deserves special attention concerning surgery, treatment and prevention. Combining regenerative medicine, tissue engineering and refined surgical techniques, will eventually lead to better clinical outcomes and improved quality of life for pediatric patients.
